# Molecular Subgroups of HRD Positive Ovarian Cancer and Their Prognostic Significance

**DOI:** 10.3390/ijms252413549

**Published:** 2024-12-18

**Authors:** Tatiana Kekeeva, Irina Dudina, Yulia Andreeva, Alexander Tanas, Alexey Kalinkin, Victoria Musatova, Natalia Chernorubashkina, Svetlana Khokhlova, Tatiana Tikhomirova, Mikhail Volkonsky, Sergey Kutsev, Dmitry Zaletaev, Vladimir Strelnikov

**Affiliations:** 1 Laboratory of Epigenetics, Research Centre for Medical Genetics, Moskvorechie st., 1, 115522 Moscow, Russia; tanas80@gmail.com (A.T.); shkarupo@mail.ru (V.M.); zalnem@mail.ru (D.Z.); vstrel@list.ru (V.S.); 2 Day Hospital No. 1, Moscow Municipal Oncological Hospital No. 62, 143515 Moscow, Russia; 3 Department of Pathology, Russian Medical Academy of Continuing Professional Education, 125993 Moscow, Russia; 4 Department of Surgical Methods of Treatment No. 9, State Budgetary Healthcare Institution Regional Oncological Dispensary, 664035 Irkutsk, Russia; 5 Oncological Department of Medical Treatment, National Medical Research Center for Obstetrics, Gynecology and Perinatology Named after V. I. Kulakov, 117997 Moscow, Russia; 6 N.N. Blokhin National Medical Research Center of Oncology, Ministry of Health of Russia, 115522 Moscow, Russia

**Keywords:** homologous recombination deficiency, BRCA1 methylation, progression-free survival, olaparib maintenance

## Abstract

Homologous recombination repair deficiency (HRD) is involved in the development of high-grade serous ovarian carcinoma (HGSOC) and its elevated sensitivity to platinum-based chemotherapy. To investigate the heterogeneity of the HRD-positive HGSOC we evaluated the HRD status, including BRCA mutations, genomic scar score, and methylation status of *BRCA1/2* genes in 352 HGSOC specimens. We then divided the HRD-positive cohort into three molecular subgroups, the BRCA mutation cohort (BRCA+), BRCA1 methylation cohort (Meth+), and the rest of the HRD+ cohort (HRD+BRCA-Meth-), and evaluated their first-line chemotherapy response, benefit from olaparib, and progression-free survival (PFS). HRD-positive status was detected in 65% (228/352) of samples. The first group, BRCA+, accounted for 45% (102/228) of HRD positive cases and showed the best outcome in platinum therapy (ORR 96%), the highest olaparib benefit (*p* = 0.006) and the highest median PFS (46 months). The frequency of the second cohort, Meth+, among HRD-positive patients was 23% (52/228). Patients with Meth+ HGSOC showed a significantly poorer outcome, with a median PFS of 19 months, a significantly lower ORR to platinum therapy (84%) and a modest, but not significant, benefit from olaparib maintenance. The third HRD+BRCA-Meth- group accounted for 32% (74/228) of HRD-positive patients and showed an ORR to platinum therapy similar to that of the BRCA+ group (90%), a higher, but not statistically significant, benefit from olaparib and a median PFS of 23 months. In conclusion, Meth+ subgroup had poor outcomes in terms of chemotherapy response, olaparib benefit, and PFS compared to the other HRD+ subgroups, requiring a more thorough follow-up.

## 1. Introduction

High-grade serous ovarian cancer (HGSOC) is the most common and fatal form of ovarian cancer [1]. Homologous recombination repair deficiency (HRD) is a molecular hallmark of approximately 58–69% of HGSOC [2,3,4,5]. HRD impairs normal DNA damage repair in tumor cells, which results in loss or duplication of chromosomal regions, termed as genomic loss of heterozygosity. HRD is remarkable for the frequent copy number alterations occurring at the whole-genome level, so quantification of large-scale structural variants is used as an indicator of the HRD phenotype [6].

Emerging clinical trials have revealed the clinical value of HRD in ovarian cancer as a predictive biomarker for poly ADP-ribose polymerase (PARP) inhibitors and first-line platinum-based chemotherapy [2,3,4,5,7]. Clinical trials PAOLA1 and PRIMA have demonstrated better progression-free survival (PFS) with Olaparib + bevacizumab than placebo + bevacizumab and niraparib than placebo in patients with HRD-positive high-grade serous or endometrioid ovarian cancer, primary peritoneal cancer, or fallopian tube cancer [2,5].

HRD can be caused by multiple factors, including germline or somatic mutations in homologous recombination-related (HRR) genes and the epigenetic inactivation of HRR genes. *BRCA1/2* germline and somatic mutations are observed approximately in 20–30% of HGSOC [2,3,4,5,6,7,8]. These mutations are predictors of platinum and PARP inhibitor responses and are prognostic factors for improved outcomes in HGSOC [2,3,4,5,7]. Another mechanism inducing HRD is *BRCA1* methylation, occurring in approximately 10–15% of HGSOC [9,10,11]. Alterations in other HRR pathway genes, including *PALB2*, *RAD51C* and *RAD51D*, have also been associated with HRD [12,13]. Therefore, HRD-positive patients represent a heterogeneous group comprising genetic, epigenetic, and unknown factors with potentially different prognoses.

Our study aimed to estimate the heterogeneity of the HRD-positive cohort by measuring genomic instability, BRCA mutations, and methylation status, and to evaluate the impact of different molecular events on clinical outcomes.

## 2. Results

### 2.1. HRD and BRCA Mutational Status

We classified 228/352 samples (65%) as HRD positive, including 102 (29%) *BRCA* mutants and 126 (36%) *BRCA* wild type cases with genomic scar score (GSS) ≥ 50 (GSS+), whereas 124 (35%) samples were identified as HRD negative (no *BRCA* mutations and GSS < 50 (GSS-)). Of note, from among 102 tumors with pathogenic or likely pathogenic *BRCA1/2* variants, 67 were located in *BRCA1* and 35 in *BRCA2*.

### 2.2. Analysis of BRCA1 and BRCA2 Methylation

**Figure 1:**
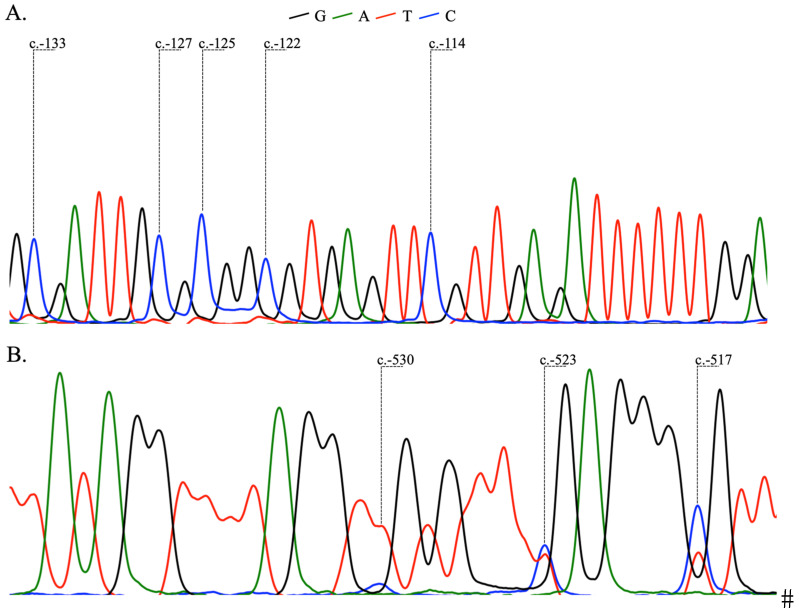
Methylated CpG dinucleotides with positions referring to the translation initiation. (**A**)—BS sequence of *BRCA1* (NM_007294.4) exon 1. (**B**)—BS sequence of *BRCA2* (NM_000059.3) promoter region.

We analyzed DNA methylation in the *BRCA1* (NM_007294.4) CpG island located in exon 1, *BRCA2* (NM_000059.3) CpG island located in exon 1, and *BRCA2* (NM_000059.3) promoter region extending 224–342 bp upstream of exon 1. The methylation frequencies were 15% for *BRCA1*, 0% for *BRCA2* in exon 1, and 8% in *BRCA2* promoter region (Table 1). The bisulfite-converted (BS) sequences of the analyzed regions are represented in Figure 1. *BRCA1* methylation located in exon 1 (hereinafter called *BRCA1* methylation) was complete (without unmethylated CpG dinucleotides) in all samples, whereas non-methylated cytosine residues were detected in *BRCA2* in all samples. None of the 102 BRCA mutated samples showed *BRCA1* methylation, confirming that *BRCA1/2* mutations and *BRCA1* methylation are mutually exclusive mechanisms of *BRCA* inactivation (*p* < 0.0001). Also, *BRCA1* methylation is exclusively associated with HRD+ HGSOC and was not found in HRD- patients (*p* < 0.0001).

We did not detect methylation of *BRCA2* in exon 1 and observed methylation of three neighboring CpG dinucleotides in the promoter *BRCA2* region in 8% of the HGSOC samples. The methylated C nucleotides are located at genomic reference sequence positions NC_000013.10:g.32889315, NC_000013.10:g.32889322 and NC_000013.10:g.32889328, i.e., 530, 523 and 517 bp upstream from the translation initiation codon of the NM_000059.3 transcript (GRCh37 assembly). No difference in *BRCA2* promoter region methylation frequency between BRCA+, BRCA-HRD+ and HRD- subgroups was observed suggesting that methylation of these CpG dinucleotides had no effect on homologous recombination repair deficiency.

### 2.3. Molecular Subgroups

Molecular analyses were performed to characterize HGSOC based on mutation, methylation, and GSS+ molecular subgroup status. Molecular subgroups were defined as the HRD-positive (HRD+) cohort, which included the BRCA mutation cohort (BRCA+), the *BRCA1* methylation cohort (Meth+), the rest of the HRD+ cohort (HRD+BRCA-Meth), and the HRD-negative (HRD-) cohort. In total, 102 (29%) HGSOC were classified as BRCA+, 52 (15%) were Meth+, 74 (21%) were HRD+BRCA-Meth-, and 124 (35%) were HRD-.

### 2.4. Association Between Molecular Subgroups and Clinical Characteristics

**Figure 2:**
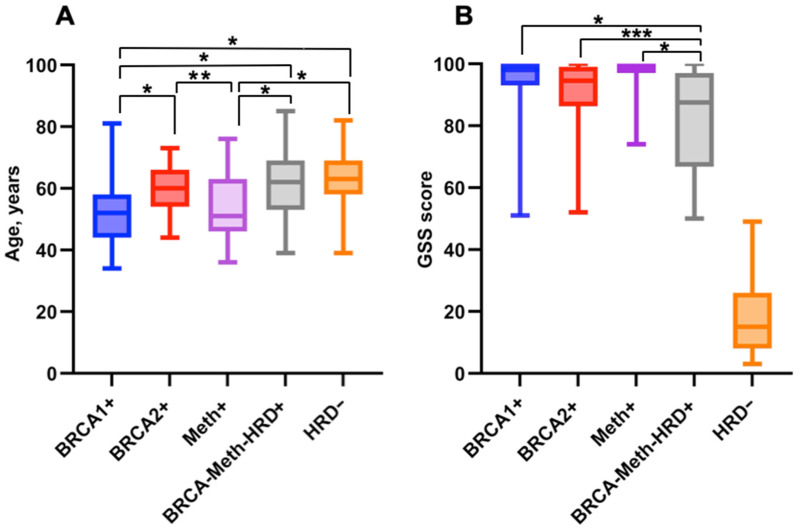
(**A**)—Distribution of patient age (years) in molecular subgroups. (**B**)—Distribution of individual GSS scores in molecular subgroups. * indicates a significant difference at *p* < 0.0001. ** indicates a significant difference at *p* = 0.006. *** indicates a significant difference at *p* = 0.01.

The median age for BRCA1+ (*n* = 67), BRCA2+ (*n* = 35), Meth+ (*n* = 52), HRD+BRCA-Meth- (*n* = 74) and HRD− (*n* = 124) cohorts were 52 [interquartile range (IQR) 44–58 years], 60 [IQR 54–66], 51 [IQR 46–63], 62 [IQR 53–69] and 63 [IQR 58–69] years old, respectively. The patients from BRCA1+ and Meth+ groups were significantly younger than patients from other molecular groups (*p* < 0.0001) (Figure 2A).

The median GSS was 98 [IQR 93–100] for BRCA1+ cohort, 94 [IQR 86–99] for BRCA2+ patients, 99 [IQR 97–100] for Meth+ patients, 87 [IQR 67–97] for the HRD+BRCA-Meth- cohort, and 16 [IQR 8–25] for the HRD- group (Figure 2B).

Clinical characteristics over molecular subgroups were available for 274 patients (Table 2). The number of first-line platinum-based chemotherapy cycles varied depending on the type of cytoreductive surgery, with an average of 3 before surgery and 3 after surgery for interval cytoreduction and with an average of 6 for primary cytoreduction. The Objective Response Rate (ORR) was documented in 90/94 (96%, 95% CI [90–98]), 27/32 (84%, 95% CI [68–84]), 52/58 (90%, 95% CI [79–95]) and 45/59 (76%, 95% CI [64–85]) of patients with BRCA+, Meth+, HRD+BRCA-Meth- and HRD-HGSOC, respectively (Table 2). The ORR differed significantly between the HRD+ and HRD- molecular subgroups (169/184 vs. 45/59, Odds Ratio (OR) = 3.5, 95% CI [1.61–7.49], *p* = 0.003) and between the BRCA+ and Meth+ molecular subgroups (90/94 vs. 27/32, OR = 4.17, 95% CI [1.12–14.15], *p* = 0.046).

The median duration of follow-up was 25 months (95% CI [21–40]) in the Meth+ group, 31 months (95% CI [26–34]) in the BRCA+ group, 26 months (95% CI [21–34]) in the HRD+BRCA-Meth- group and 30 months (95% CI [18–42]) in the HRD- group.

**Figure 3:**
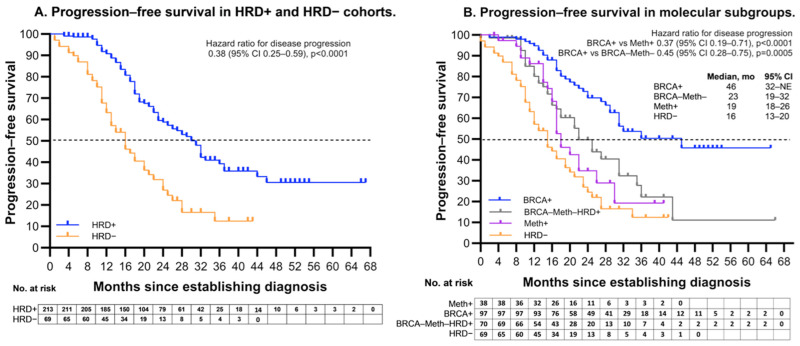
Progression free survival. (**A**)—PFS in patients with HRD- and HRD+ HGSOC. (**B**)—PFS within HRD positive cohort, which included the BRCA mutation cohort (BRCA+), the *BRCA1* methylation cohort (Meth+), all the rest of HRD+ cohort (BRCA-Meth-). NE—not estimated.

The PFS among HRD positive patients was significantly longer than that of HRD negative patients, median PFS was 31 months (95% CI [24–32]) and 16 months (95% CI [13–20]), respectively (Hazard ratio (HR) 0.38; 95% CI [0.25–0.59]; *p* < 0.0001) (Figure 3A). Among the HRD + subgroups, BRCA+ HGSOC showed superior outcomes, with a median PFS of 46 months (95% CI [32–NE (not estimated]). HRD+BRCA-Meth- patients had a median PFS of 23 months (95% CI [19–32]). In contrast, the Meth+ patients had a median PFS of 19 months (95% CI [18–27]). The PFS among BRCA+ HGSOC was significantly better than that of Meth+ patients (HR 0.37; 95% CI [0.19–0.71]; *p* < 0.0001) and that, of HRD+BRCA-Meth- patients (HR 0.45; 95% CI [0.28–0.75]; *p* = 0.0005) (Figure 3B). 

The multivariable Cox proportional hazards analysis confirmed that cytoreductive surgery without residual macroscopic disease, timing of cytoreductive surgery, response to first-line chemotherapy, BRCA mutation status, and olaparib maintenance were in-dependent prognostic factors for PFS in patients with HGSOC (Table 3).

**Figure 4:**
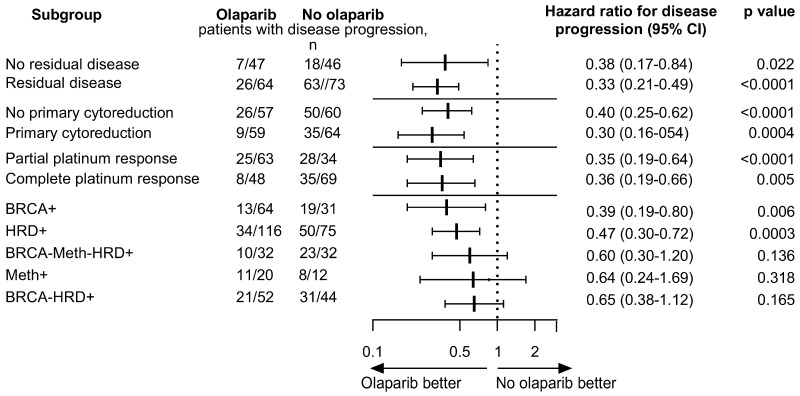
Forest plot of hazard ratios comparing progression-free survival (PFS) of patients treated with chemotherapy + olaparib with that of patients treated with chemotherapy only.

Analyses of PFS in subgroups defined according to independent prognostic factors and molecular subgroups of patients treated with chemotherapy + olaparib versus chemotherapy only are shown in Figure 4. In the HRD-positive patients, olaparib provided a significant clinical benefit over placebo with respect to the median PFS in patients with BRCA+ (median was not reached vs. 30 months; HR 0.39, *p* = 0.006). Assessment in the olaparib and control groups in the subgroup of Meth+ and HRD+BRCA-Meth- patients showed median PFS 19 versus 17 months (HR 0.64, *p* = 0.17) and 32 versus 23 months (HR 0.6, *p* = 0.1), respectively. Our result of subgroup analysis according to molecular status demonstrated that olaparib could improve the PFS in all HRD positive groups, although the significant magnitude of benefit appeared in patients with BRCA+ tumors only.

## 3. Discussion

The identification of patients with diverse prognoses has substantial clinical significance. Emerging clinical trials have revealed the clinical value of HRD in ovarian and breast cancers as a predictive biomarker for PARP inhibitors and first-line platinum-based chemotherapy. Among first-line adjuvant chemotherapy patients, the HRD status significantly influenced PFS and overall survival [14]. In our study, the PFS of HRD-positive patients was significantly longer than that of HRD-negative patients, with a median PFS of 31 months and 16 months, respectively.

The ORR to platinum therapy also differed significantly when the HRD+ subgroup was compared to the HRD- subgroup (92% vs. 76%, OR = 3.5, 95% CI [1.61–7.49], *p* = 0.003). However, limited research has documented platinum-based treatment predictions using HRD as a biomarker in patients with ovarian cancer. Feng et al. investigated the association between HRD status and response to platinum-based chemotherapy in 240 Chinese patients with HGSOC. Platinum-sensitive patients had higher HRD scores than platinum-resistant patients; however, the difference was not significant (*p* = 0.086). The platinum sensitivity rate was higher in HRD+BRCA+ (97%) and HRD+BRCA- tumors (90%) than in HRD-tumors (74%). The authors also found that platinum-sensitive patients tended to have *BRCA1/2* or other HRR gene mutations [15]. The TNT trial investigators assessed carboplatin and docetaxel in advanced triple negative breast cancer. Patients with germinal BRCA mutations had a significantly better ORR to carboplatin than docetaxel (68% vs. 33%). In contrast, patients with *BRCA1* methylation and HRD+ status did not benefit from platinum therapy [16].

To evaluate the molecular heterogeneity of the HRD-positive group, we divided the HRD-positive patients into three subgroups. The first subgroup, the *BRCA* mutation cohort, had the most favorable outcome in terms of platinum response (ORR, 96%), the best olaparib benefit (HR 0.39), and the highest median PFS (46 months in total BRCA+ subroup). Of note, the median PFS for BRCA+ patients who did not receive olaparib, was 30 months and median PFS was not reached for olaparib-treated BRCA+ patients due to insufficient observation time (31 months of median follow-up). In the SOLO1 trial, the median PFS for BRCA+ olaparib-treated patients was 56 months with longer-term follow-up of 58 months [17].

The second subgroup, with *BRCA1* methylation, and the third one (HRD+BRCA-Meth-) were both enriched in high-GSS HGSOC (36% in total). Of note, in neither of these three groups, there were any HGSOC with GSS low. The patients with *BRCA1* methylation as well as *BRCA1* mutation were significantly younger than patients with *BRCA2* mutations and patients from HRD+BRCA-Meth- and HRD- groups (*p* < 0.0001). The frequency of *BRCA1* methylation among HRD-positive patients was 23% (52/228). *BRCA1* epigenetic silencing in tumors is assumed to have the same effect as *BRCA1* mutations in terms of sensitivity to platinum chemotherapy and PARP inhibitors, and survival outcomes [18,19]. In our data, the ORR to platinum was significantly lower in the Meth+ molecular subgroup than in the BRCA+ subgroup (84% vs. 96%, *p* = 0.046). The subgroup of Meth+ patients showed hazard ratio of 0.64 (95% CI [0.24 to 1.69], *p* = 0.3) and the modest not statistically significant benefit from olaparib maintenance (median PFS 19 months with olaparib versus 17 months without olaparib, HR 0.64, *p* = 0.17). Also, *BRCA1* methylation HGSOC cohort exhibited poor survival, with a median PFS, similar to that of *HRD*-negative cases. Consistently, the TCGA data showed that patients with epigenetically silenced *BRCA1* had survival rates similar to *BRCA1/2* wild-type HGSOC [20]. Our findings are also in line with those of Takaya et al., who demonstrated that *BRCA1* and *RAD51C* methylation is a poor prognostic subtype among HRD-positive cases in terms of PFS and overall survival [10]. In our data, the *BRCA1* methylation is associated with HRD+ HGSOC and was not found in BRCA+ patients (*p* < 0.0001). This suggests that *BRCA1* is inactivated by mutually exclusive genetic and epigenetic mechanisms and that patient prognosis depends on the mechanism of inactivation.

Occurrence of platinum-based chemotherapy resistance is associated with several mechanisms, including tumor heterogeneity, reduced drug concentration to the target, alteration in drug target structure, increased repair of the lesions induced, in particular restoring of the HRR function [21,22]. It can be assumed that after platinum-based therapy, the secondary loss of *BRCA1* methylation occurs faster than secondary resistance genetic changes, such as the reversion mutation in *BRCA1/2*-mutated cases [12,21]. Patch et al. described the case of a patient initially diagnosed with *BRCA1* methylated, platinum sensitive primary HGSOC who developed recurrent cancer that was non-methylated and platinum-resistant [21]. Analysis of *BRCA1*-methylated platinum-sensitive recurrent HGSOC confirmed that methylation loss can occur after chemotherapy treatment [23]. Similarly, HGSOC exhibits monoallelic methylation of *RAD51C* and elevated *RAD51C* gene expression (compared to controls with biallelic methylation of *RAD51C*) after first-line chemotherapy [24]. The platinum-based therapy of *BRCA1* methylated triple negative breast cancer patients resulted in allelic loss of *BRCA1* methylation, increased BRCA1 expression, and platinum resistance [25,26]. *BRCA1*-methylated triple negative breast cancer-derived xenografts that had been treated by neoadjuvant chemotherapy responded poorly to olaparib, similarly to wild-type *BRCA1*. In contrast, treatment-naive BRCA1-deficient xenografts (*BRCA1*-methylated and *BRCA1*-mutated) well responded to olaparib [25]. These data suggest that, unlike BRCA-mutated tumors, where BRCA loss is a genetically stable structural state, *BRCA1* methylated tumors are easily reversible and highly adaptive to chemotherapy as they can quickly restore the BRCA1 expression and become resistant to platinum and PARP inhibitors. One hypothesis for the loss of *BRCA1* methylation following chemotherapy is the substitutive expansion of a non-methylated subclone from a heterogeneous tumor cell population. Another hypothesis is that the non-methylated subclone derives from the same genomic origin as its *BRCA1* methylated primary ancestor and eventually loses *BRCA1* methylation after treatment [26]. Currently, no consensus has been reached regarding the effect of *BRCA1* methylation on the survival of patients with HGSOC, and many studies have not supported our findings. While some studies have reported statistically significant improvements in survival compared to non–*BRCA1*-methylated HGSOC, others have observed trends toward a worse outcome [9,11].

The third molecular group, the HRD+ cohort without BRCA mutations or *BRCA1* methylation, had an objective response rate to platinum therapy similar to that of the BRCA+ group (ORR, 90%). The HRD+BRCA-Meth- group showed a higher, but not statistically significant, benefit from olaparib (median PFS 32 months with olaparib versus 23 months without olaparib, HR 0.6) and a longer PFS than *BRCA1* methylation group. The HRD+BRCA-Meth- cohort had the lowest median GSS value (87 (IQR 67–97)) among all HRD-positive cases. It is known that next-generation sequencing restricted to exons and exon-intron boundaries, cannot identify some pathogenic variants, such as large rearrangements, indels of more than 35 bp and deep intronic variants [27,28]. Also, NGS coverage is not uniform and is varied in different panels and may be underrepresented in variants with low allelic frequency [29]. Hence, we hypothesized that the HRD+BRCA-Meth- group may contain an unknown proportion of BRCA+ patients with improved prognosis.

It is supposed that the GSS scores can indicate the degree of severity of homologous recombination deficiency. Su et al. showed that in 342 HRD-positive HGSOC cases with *BRCA1/2* mutations, a higher tumor stage correlated with higher HRD scores, with the score elevating from stage I to III and slightly declining at stage IV [30]. In our study, the highest GSS were observed in the *BRCA1* methylation group. 

In conclusion, we showed that *BRCA1* methylation subgroup had poor outcomes in terms of chemotherapy response, olaparib benefit, and PFS compared to the other HRD+ subgroups. Our data suggest the importance of *BRCA1* methylation testing for identifying patients with a worse prognosis, who require a more thorough follow-up.

## 4. Material and Methods

### 4.1. Study Cohort

The tumor samples of 352 patients with histologically confirmed new diagnosis (of high-grade (stage III or IV of FIGO classification 2014) serous ovarian, primary peritoneal, and/or fallopian tube cancer recruited from Russian hospitals between 2019 and 2022 were analyzed. The eligible patients were women aged 18 years or older who were willing to provide written informed consent for participation in the study. Patients with endometrioid, serous low-grade, mucinous, clear-cell, undifferentiated, or recurrent serous high-grade carcinomas were excluded.

### 4.2. Histopathological Specimens

Formalin-Fixed Paraffin-Embedded (FFPE) tumor tissue blocks were obtained before neoadjuvant/adjuvant chemotherapy within 120 days of enrolment from the primary debulking surgery or primary tumor biopsy (if interval cytoreduction was performed). The histological subtype and tumor, node, and metastasis stages were reviewed by a pathologist.

### 4.3. Outcomes

The cases were assessed locally in hospitals. Tumor assessments (complete response, partial response, stable disease, and progressive disease) were performed after the last chemotherapy cycle according to the Response Evaluation Criteria in Solid Tumors, version 1.1. We brought the patients who had no residual disease after primary surgery and remained NED (no evidence of disease) and patients who achieved a complete response together. The Objective Response Rate (ORR) was defined as the percentage of patients who had a partial response or complete response to the treatment. Data on hospital-assessed progression were collected until progression or loss to follow-up in 274 patients.

### 4.4. Sample Preparation

Manual macrodissection was performed on each selected sample. We used only the specimens where stromal cell contamination was lower than 50%. The slides were prepared as follows: we cut 5-micron section on uncharged slide, provided hematoxylin and eosin staining and collected tissue from tumor area. The number of slides varied from 5 to 10 depending on the tumor area. DNA was isolated using a GeneRead DNA FFPE Treatment Kit (Qiagen).

### 4.5. Analysis of CpG Island Methylation

Bisulfite-converted DNA was obtained using the EpiTect Fast FFPE Bisulfite Kit (Qiagen, Hilden, Germany). Bisulfite conversion and subsequent purification were performed according to manufacturer’s instructions. CpG island methylation was assayed using bisulfite Sanger sequencing. The primers and annealing temperatures used for hot-start PCR are listed in Table 4. Sanger sequencing was performed using the BigDye Terminator Cycle sequencing Kit (Applied Biosystems, Waltham, MA, USA). Chromatograms obtained from bisulfite sequencing were analyzed using the SeqBase software (http://www.epigenetic.ru/projects/seqbase, accessed on 15 December 2024).

### 4.6. NGS Analysis

HRD assessment was performed using the HRD Focus Assay (AmoyDx, Xiamen, China) following the manufacturer’s instructions, as described previously [8]. This assay allowed the simultaneous analysis of SNVs and indels in the whole coding regions and exon-intron boundaries of BRCA1/BRCA2 and estimated a genomic scar score (GSS) based on the analysis of 24,000 SNPs. A GSS equal or higher than 50 was indicative of HRD positivity. The bioinformatic algorithm applied for the NGS data analysis was Andas AmoyDx (version 1.1.1). The GSS algorithm has been previously described [31].

### 4.7. Statistical Analysis

The normality of the distribution of continuous variables was established using the Kolmogorov-Smirnov test. Non-normal variables (age and GSS) are reported as median [IQR]. Statistical analyses were performed for four groups: the HRD-positive (HRD+) cohort, which included the *BRCA* mutation cohort (BRCA+); the *BRCA1* methylation cohort (Meth+); the rest of the HRD+ cohort (HRD+BRCA-Meth-); and the HRD-negative cohort (HRD-). PFS in each group was estimated using the Kaplan–Meier method. Hazard ratios were estimated using the log-rank method. The ORR confidence interval was calculated by Wilson/Brown method. Fisher’s exact test was used to compare ORR between molecular subgroups. Nonparametric t-test was used to compare the variables that had a non-normal distribution. *p*-values < 0.05 were considered statistically significant. Median follow-up time was calculated by reverse Kaplan-Meier analysis. Descriptive statistics, forest plots, Fisher’s exact test and Kaplan–Meier methods were performed using GraphPad Prism 10.0.3. Cox proportional hazards analysis was performed using XLSTAT 2019 software.

## Figures and Tables

**Table 1:** The *BRCA1* and *BRCA2* methylation frequencies.

	Number of Patients (%)		
HGSOC Samples	*BRCA1* Exon1 Methylation	*BRCA2* Exon1 Methylation	*BRCA2* Promoter Methylation
All	52/352 (15%)	0/352	28/352 (8%)
BRCA+	0/102	0/102	10/102 (10%)
BRCA-/HRD+	52/126 (42%)	0/126	10/126 (8%)
HRD-	0/124	0/124	7/124 (6%)

**Table 2:** Clinical characteristics of patients.

	Molecular Subgroups			
	HRD+			HRD−
Clinical characteristic	BRCA+ (*n* = 97)	Meth+ (*n* = 38)	BRCA–Meth– (*n* = 70)	GSS– (*n* = 69)
Age				
Mean	56	52	62	65
Range	32–82	36–76	39–85	39–82
Stage				
III	65/97 (67%)	28/38 (74%)	47/70 (67%)	43/69 (62%)
IV	32/97 (33%)	10/38 (26%)	23/70 (33%)	26/69 (38%)
Timing of cytoreductive surgery				
Primary debulking surgery	48/95 (51%)	17/31 (55%)	35/57 (61%)	23/57 (40%)
Interval cytoreduction	37/95 (39%)	10/31 (32%)	12/57 (21%)	21/57 (37%)
Not perfomed	10/95 (10%)	4/31 (13%)	10/57 (18%)	13/57 (23%)
Unknown	2	7	13	12
Cytoreductive surgery outcome				
Cytoreductive surgery without residual macroscopic disease	38/92 (41%)	14/31 (45%)	20/48 (42%)	21/59 (36%)
Cytoreductive surgery with residual macroscopic disease	44/92 (48%)	13/31 (42%)	18/48 (37%)	25/59 (42%)
No cytoreductive surgery	10/92 (11%)	4/31 (13%)	10/48 (21%)	13/59 (22%)
Unknown	5	7	22	10
Numbers of cycles of first-line platinum-based chemotherapy				
1–5	5/88 (6%)	2/24 (8%)	8/61 (13%)	15/67 (22%)
6	79/88 (90%)	21/24 (88%)	50/61 (78%)	47/67 (70%)
7–8	4/88 (4%)	1/24 (4%)	3/61 (5%)	5/67 (8%)
Unknown	9	14	9	2
Disease response to first-line chemotherapy				
Complete response/NED	43/94 (46%)	17/32 (53%)	33/58 (57%)	24/59 (40%)
Partial response	47/94 (50%)	10/32 (31%)	19/58 (33%)	21/59 (36%)
Stable disease	4/94 (4%)	2/32 (6%)	6/58 (10%)	7/59 (12%)
Progressive disease	0	3/32 (10%)	0	7/59 (12%)
Unknown	3	6	12	10
Response chemotherapy rates				
Disease control rate	94/94 (100%)	29/32 (90%)	58/58 (100%)	52/59 (88%)
Objective response rate	90/94 (96%)	27/32 (84%)	52/58 (90%)	45/59 (76%)
PARP-inhibitors maintenance therapy				
Yes	64/95 (67%)	20/32 (63%)	32/64 (50%)	3/63 (5%)
No	31/95 (33%)	12/32 (37%)	32/64 (50%)	60/63 (95%)
Unknown	2	6	6	6

**Table 3:** Univariable and multivariable Cox proportional hazards regression analysis of factors affecting PFS. HR—hazard ratio, CI—confidence interval, CR—complete re-sponse, PR—partial response, SD—stable disease, PD—progressive disease.

Variables	Univariable		Multivariable	
	*p* value	HR (95% CI)	*p* value	HR (95% CI)
Age (≥60 vs. <60 years)	**0.008**	1.70 (1.15–2.52)	0.69	1.10 (0.69–1.74)
FIGO stage (IV vs. III)	**0.007**	1.70 (1.15–2.49)	0.87	1.04 (0.67–1.60)
BRCA1 methylation (Meth+ vs. Meth-)	0.15	1.45 (0.87–2.42)	0.48	1.24 (0.68–2.25)
BRCA mutation (BRCA+ vs. BRCA-)	**<0.0001**	0.34 (0.00–0.53)	**0.002**	0.45 (0.00–0.75)
Olaparib maintence (olaparib vs. no olaparib)	**<0.0001**	0.39 (0.00–0.58)	**0.003**	0.50 (0.00–0.79)
Timing of cytoreductive surgery (primary vs. non-primary)	**<0.0001**	0.40 (0.00–0.61)	**0.004**	0.52 (0.00–0.82)
Residual disease (no residual vs. residual)	**<0.0001**	0.36 (0.00–0.56)	**0.001**	0.43 (0.00–0.69)
Chemotherapy response (CR/PR vs. SD/PD)	**<0.0001**	0.33 (0.00–0.53)	**0.01**	0.52 (0.00–0.86)

**Table 4:** Bisulfite sequencing PCR primers for the analysis of *BRCA1* and *BRCA2* gene methylation.

Gene	Primer (5′ to 3′)	Annealing, °C
*BRCA1* exon1 F *BRCA1* exon1 R	GTATTTTGAGAGGTTGTTGTTTAG TACCTTTACCCAAAACAAAAAATAAA	62
*BRCA2* exon1 F *BRCA2* exon1 R	GGTTTATTTAGGTTTGATTTT ATCACAAATCTATCCCCTCAC	60
*BRCA2* promoter F *BRCA2* promoter R	TTGGGGAATAGGTTTTGAGAGAATATTT AATCCCAAACCACCCTACTTAAAAAAAC	62

## Data Availability

Research data may be provided upon reasonable request.
